# A20 Restrains Thymic Regulatory T Cell Development

**DOI:** 10.4049/jimmunol.1602102

**Published:** 2017-08-25

**Authors:** Julius Clemens Fischer, Vera Otten, Maike Kober, Christoph Drees, Marc Rosenbaum, Martina Schmickl, Simon Heidegger, Rudi Beyaert, Geert van Loo, Xian Chang Li, Christian Peschel, Marc Schmidt-Supprian, Tobias Haas, Silvia Spoerl, Hendrik Poeck

**Affiliations:** * Klinik und Poliklinik für Innere Medizin III, Klinikum rechts der Isar, Technische Universität, 81675 Munich, Germany; † Institut für Klinische Chemie und Pathobio-chemie, Klinikum rechts der Isar, Technische Universität, 81675 Munich, Germany; ‡ Department of Biomedical Molecular Biology, Ghent University, B-9052 Ghent, Belgium; § Inflammation Research Center, VIB, B-9052 Ghent, Belgium; ¶ Immunobiology & Transplant Science Center, Houston Methodist Hospital, Texas Medical Center, Houston, TX 77030; ‖ Department of Surgery, Weill Cornell Medical College of Cornell University, New York, NY 10065

## Abstract

Maintaining immune tolerance requires the production of Foxp3-expressing regulatory T (T_reg_) cells in the thymus. Activation of NF-κB transcription factors is critically required for T_reg_ cell development, partly via initiating Foxp3 expression. NF-κB activation is controlled by a negative feedback regulation through the ubiquitin editing enzyme A20, which reduces proinflammatory signaling in myeloid cells and B cells. In naive CD4^+^ T cells, A20 prevents kinase RIPK3-dependent necroptosis. Using mice deficient for A20 in T lineage cells, we show that thymic and peripheral T_reg_ cell compartments are quantitatively enlarged because of a cell-intrinsic developmental advantage of A20-deficient thymic T_reg_ differentiation. A20-deficient thymic T_reg_ cells exhibit reduced dependence on IL-2 but unchanged rates of proliferation and apoptosis. Activation of the NF-κB transcription factor RelA was enhanced, whereas nuclear translocation of c-Rel was decreased in A20-deficient thymic T_reg_ cells. Furthermore, we found that the increase in T_reg_ cells in T cell–specific A20-deficient mice was already observed in CD4^+^ single-positive CD25^+^ GITR^+^ Foxp3^−^ thymic T_reg_ cell progenitors. T_reg_ cell precursors expressed high levels of the tumor necrosis factor receptor superfamily molecule GITR, whose stimulation is closely linked to thymic T_reg_ cell development. A20-deficient T_reg_ cells efficiently suppressed effector T cell–mediated graft-versus-host disease after allogeneic hematopoietic stem cell transplantation, suggesting normal suppressive function. Holding thymic production of natural T_reg_ cells in check, A20 thus integrates T_reg_ cell activity and increased effector T cell survival into an efficient CD4^+^ T cell response.

Tcell–mediated immune tolerance requires induced and naturally derived regulatory T (T_reg_) cells, the latter generated during thymic T cell selection. Foxp3 is a master transcription factor for the development and function of T_reg_ cells, and defective Foxp3 expression results in severe autoimmune phenotypes in mice and men (1, 2). Although the regulation of naturally derived T_reg_ cell development is still incompletely understood (3), it is clear that TCR stimulation along with signals from common γ-chain (γc) receptor–linked cytokines IL-2 and IL-7 are essential to induce Foxp3 expression and T_reg_ cell development (4). Upon TCR engagement, protein kinase C and the Carma1/Bcl10/Malt1 protein complex are recruited to finally induce NF-κB transcription factor activity, key regulator of lymphocyte differentiation, expansion, activation, and survival (5, 6). Mice bearing defects in the TCR signaling pathway (including TAK1, Bcl10, CARMA1, protein kinase C u, and IKK2) show selective impairments in development and function of T_reg_ cells, whereas conventional T cell development seems to be less affected (7–12). Furthermore, mice deficient for γc receptors, which transmit signaling initiated by homeostatic cytokines such as IL-2 and whose expression is regulated by various mechanisms including the NF-κB pathway, also lack T_reg_ cells (13–15). The NF-κB transcription factor c-Rel is highly expressed in thymic T_reg_ cells and directly promotes transcription of Foxp3 in the thymus. Accordingly, T_reg_ cell numbers are strongly reduced in the absence of the NF-κB family proteins p50 and c-Rel (16–18). One of the key regulators of both NF-κB activation and TCR signaling is the ubiquitin editing enzyme A20, which limits NF-κB signaling after activation by TNF, IL-1/TLRs, and the TCR (19). Consistent with this, A20-deficient mice are hypersensitive to LPS and TNF exposure, and die perinatally because of severe inflammation and multiorgan failure (20). Lineage-specific A20 deficiency in various cell types such as B cells, dendritic cells, intestinal epithelial cells, and hepatocytes results in autoimmunity, higher susceptibility to inflammatory diseases, or hepatocellular carcinoma (21–25), and clinical studies link genetic A20 polymorphisms to human autoimmune and lymphoproliferative disorders (26–30). In T cells, TCR activation and Carma1/Bcl10/Malt1 complex formation is followed by K63-linked polyubiquitination of MALT1, resulting in IκB kinase complex activation and NF-κB signaling. A20 cleaves the polyubiquitin chains from MALT1, thus suppressing NF-κB activation. In return, MALT1 also has a proteolytic activity, which can inactivate A20 (31, 32). In CD8^+^ T cells, A20 deletion leads to sustained expression of the NF-κB family members c-Rel/RelA and increased production of proinflammatory cytokines such as IFN-γ, TNF, and IL-2 (33). In CD4^+^ T cells, A20 is essential for survival and expansion by promoting autophagy and protecting from necroptotic cell death (34, 35). Intriguingly, unrestricted necroptosis in A20-deficient CD4^+^ cells affects both the Th1 and the Th17 compartment, leading to reduced inflammation in a CD4^+^ T cell–dependent model of autoimmune encephalomyelitis (34). In NKT cell sub-lineages NKT1 and NKT2, A20 was also shown to restrict TCR-dependent activation and survival, thereby controlling NKT cell differentiation (36). However, the role of A20 for T_reg_ cell differentiation, central modulators of inflammatory responses in vivo, remains unexplored. In this article, we demonstrate that A20 regulates the de novo generation of naturally derived T_reg_ cells in the thymus in a cell-intrinsic fashion independent of γc-cytokine IL-2 signaling. This developmental advantage could be attributed to enhanced emergence of thymic T_reg_ cell progenitors. Importantly, the functionality of A20-deficient T_reg_ cells is unchanged in vitro and in the prevention of lethal allogeneic T cell activity in a preclinical model of graft-versus-host disease (GVHD).

## Materials and Methods

### Animals

Mice with cell type–specific deficiency of A20 in T cells were generated by breeding *A20*^F/F^ mice (25) with CD4Cre transgenic mice (37) as previously described (38). C57BL/6 (H-2Kb, Thy-1.2) and BALB/c (H-2Kd, Thy-1.2) mice were purchased from Janvier Labs. Mice were between 6 and 12 wk of age at the onset of experiments if not indicated otherwise. Animal protocols were approved by the local regulatory agency (Regierung von Oberbayern, Munich, Germany).

### Allogeneic hematopoietic stem cell transplantation and GVHD

Allogeneic bone marrow (BM) transplantations were performed as previously described (39). In brief, female, 6- to 8-wk-old BALB/c recipients were lethally irradiated with total body irradiation (TBI; 2 × 4.5 Gy). Directly after the second irradiation step mice received 5 × 10^6^ T cell– depleted C57BL/6 wild type (WT) BM cells for reconstitution. T cell depletion of freshly isolated BM cells was performed using CD90.2 microbeads (Miltenyi Biotec). In addition, some mice received 2 × 10^5^ CD8^+^ T cells and 2 × 10^5^ CD4^+^ CD25^−^ effector T cells with or without 6 × 10^5^ CD4^+^ CD25^+^ T_reg_ cells as indicated in the figure legends. All T cell subsets were isolated from complete splenocytes using the T_reg_ cell isolation kit (Miltenyi Biotec) and the CD8 T cell isolation kit (Miltenyi Biotec) according to the manufacturer's instructions.

### Syngeneic hematopoietic stem cell transplantation and competitive T cell development

CD45.1^+^ CD45.2^+^ (double-positive) C57BL/6 WT mice were used for competitive T cell development experiments. Conditioning therapy of C57BL/6 recipient mice before the syngeneic BM transplantation was performed as previously described (40). After conditioning therapy (2 × 5.5 Gy), recipient mice received 2.5 × 10^6^ T cell–depleted C57BL/6 WT CD45.1^+^ CD45.2^−^ BM cells. In addition, recipient mice received either 2.5 × 10^6^ A20^F/F^ CD4^cre−^ or 2.5 × 10^6^ A20^F/F^ CD4^cre+^ T cell–depleted C57BL/6 BM cells, expressing only CD45.2, but not CD45.1. Mice were analyzed 3 mo after transplantation. Exact ratios of transplanted and engrafted CD45.1^+^ and CD45.2^+^ BM cells were determined by analysis of CD45.1 and CD45.2 expression of CD4^−^ CD8^−^ cells. The engraftment ratio was calculated for all transplanted animals separately. Further analysis of cell subsets was normalized to this ratio.

### Flow cytometry and Abs

All Abs were purchased from eBioscience, BioLegend, and Miltenyi Biotec. For cell viability analysis, a near-infrared Live/Dead reagent (Molecular Probes; Thermo Fisher Scientific) was used according to the manufacturer's instructions. The following Abs were used for cell surface or intracellular staining and flow cytometric analysis: CD4 (GK 1.5/RM4-5), CD8 (53-6.7), CD25 (PC61.5), CD44 (IM7), CD62L (MEL-14), c-Rel (REA397), Foxp3 (FJK16s), GITR (DTA-1), and KI-67 (16A8). Cells were stained in PBS supplemented with 2.5% BSA using fluorochrome-conjugated Abs against specific cell surface markers in different dilutions for 20 min at 4°C. For Foxp3 staining, the Foxp3/transcription factor staining buffer set (eBioscience) was used according to the manufacturer's instructions. Active caspase-3 stain was performed directly before further viability and cell surface staining using the CaspGLOW Fluorescein Active Caspase-3 Staining Kit (Thermo Fisher Scientific) according to the manufacturer's instructions. In vivo cell proliferation was analyzed using the Click-iT Plus EdU (5-ethynyl-2′-deoxyuridine) Alexa Fluor 647 Flow Cytometry Assay Kit (Thermo Fisher Scientific) according to the manufacturer's instructions. The assay was performed 24 h after i.p. injection of EdU. Treatment dose of EdU (50 mg/kg per mouse) was used as previously described (41). Analyses of cell populations were performed by flow cytometry with a FACSCanto II (BD). Data analysis was performed using FlowJo software (Tree Star). Flow cytometry sorting was performed with a FACSAria III (BD Biosciences). The anti-mouse IL-2 Ab (JES6-1A12; BioXCell) was used to neutralize IL-2 in vivo. In indicated experiments, 500 mg of anti–IL-2 was injected i.p. 6 and 3 d before analysis.

### Imaging flow cytometry

Thymocytes were subjected to MACS-based depletion of CD8^+^ T cells (CD8 microbeads; Miltenyi Biotec). Cells were then either left untreated or were stimulated with 100 nM PMA (Sigma) and 1 mM ionomycin (Calbiochem) for 30 min at 37°C, 5% CO_2_. Extracellular and intracellular Foxp3 staining was performed as described earlier. Intracellular c-Rel staining was performed overnight. DAPI (Thermo Fisher Scientific) was applied as a nuclear stain subsequent to Ab stainings. Stained cell suspensions were acquired on an ImageStreamX Mark II (Amnis; Merck Millipore) imaging flow cytometer. Data analysis was performed using the wizard for nuclear translocation of the IDEAS software (Amnis), where determination of nuclear translocation is based on a similarity score that quantifies the correlation of nuclear stain and translocation probe intensities. High correlation and thus high similarity scores represent strong nuclear translocation, whereas low scores are indicative of cytoplasmic localization (42).

### Phospho–NF-κB p65 (S536) phosphoflow

Cells were either left untreated or were stimulated with 100 nM PMA (Sigma-Aldrich) and 1 mM ionomycin (Calbiochem) for 30 min at 37°C, 5% CO_2_. After washing the cells with PBS, live/dead cell staining was performed with a fixable viability dye (eBioscience) followed by surface staining. Cells were fixed with 2% PFA for 40 min, permeabilized by two washes with Perm/Wash buffer (eBioscience), and stained overnight with anti-Foxp3 and anti–phospho-NFKBp65 (Ser536) (93H1; Cell Signaling) Abs. After two washes with Perm/Wash buffer, cells were stained with an allophycocyanin-labeled anti-rabbit IgG Ab (A10931; Invitrogen).

### Isolation of CD4^+^ CD25^+^ T_reg_ cells with magnetic cell sorting

CD4^+^ CD25^+^ T_reg_ cells were isolated from splenic single-cell suspensions using the MACS T_reg_ cell isolation kit (Miltenyi Biotec) according to the manufacturer's instructions.

### T_reg_ cell suppression assay

MACS-sorted CD4^+^ CD25^−^ T effector cells were cultured in RPMI 1640 medium in a 96-well plate at a concentration of 1 × 10^5^ cells per well. Soluble anti-CD3 (1 μg/ml; clone 2C11; BD Pharmingen) and irradiated APCs were used in a 1:2 ratio. APCs as stimulator cells were prepared from syngeneic mice by depleting the CD4^+^ T cell fraction using anti-CD4 microbeads according to the manufacturer's instructions (Miltenyi Biotec). For suppression, CD4^+^ CD25^+^ MACS-sorted A20^F/F^ CD4^cre+^ or A20^F/F^ CD4^cre−^ T_reg_ cells were added at the indicated ratio to A20^F/F^ CD4^cre−^ T effector cells. Proliferation of CD4^+^ CD25^−^ T effector cells was analyzed using the CellTrace Violet Cell Proliferation Kit (Invitrogen) according to the manufacturer's instructions. The percentage of proliferating cells was analyzed after 72 h.

### T_reg_ cell expansion and differentiation of naive CD4^+^ T cells in vitro

#### T_reg_ cell expansion

T_reg_ cells (CD4^+^ CD25^+^) were prepared by FACS or MACS isolation. Cells were activated with anti-murine CD3 (1 μg/ml; clone 17A2; BioLegend) plus soluble anti-murine CD28 (1 μg/ml; clone 37.51; BioLegend) with or without 50 international units human IL-2 (PeproTech) after precoating of 96-well tissue culture plates (Sigma-Aldrich) with 10 μg/ml F(ab′)_2_ fragment of IgG H+L (Jackson ImmunoResearch). In indicated experiments, cells were activated with soluble beads preloaded with CD3 and CD28 Abs according to the manufacturer's instructions (130-095-925; Miltenyi Biotec). At indicated time points after activation (days 4 and 8), intracellular Foxp3 was stained and T_reg_ cells were counted. Cell number was determined with flow cytometry cell counting beads according to the manufacturer's instructions (Thermo Fischer Scientific).

#### Differentiation of naive CD4^+^ T cells

Naive CD4^+^ T cells (CD62L^+^ CD44^−^) were prepared by FACS; sorted cells were activated with anti-murine CD3 (0.2 μg/ml) plus soluble anti-murine CD28 (1 μg/ml). Generation of induced T_reg_ (iT_reg_) cells was performed in the presence of 10 ng/ml human TGF-β1 (R&D Systems) with or without 50 international units human IL-2. CD4^+^ T cells were cultured under polarizing conditions (for 4 d), and cell polarization was assessed by intracellular Foxp3 staining. Cell number was determined as described above.

### Statistics

GraphPad Prism version 6 was used for statistical analysis. Survival was analyzed using the log-rank test. Differences between means of experimental groups were analyzed using the one- or two-tailed unpaired Student *t* test, corresponding to the distribution shape of our observations. We used ordinary one-way ANOVA for multiple comparisons and always performed Dunnett's test for multiple-test corrections. The applied statistical tests are indicated in the figure legends. For visual clarity, data are shown as mean ± SEM throughout.

## Results

### Increased numbers of T_reg_ cells in mice with T cell–specific A20 deletion

**Figure 1:**
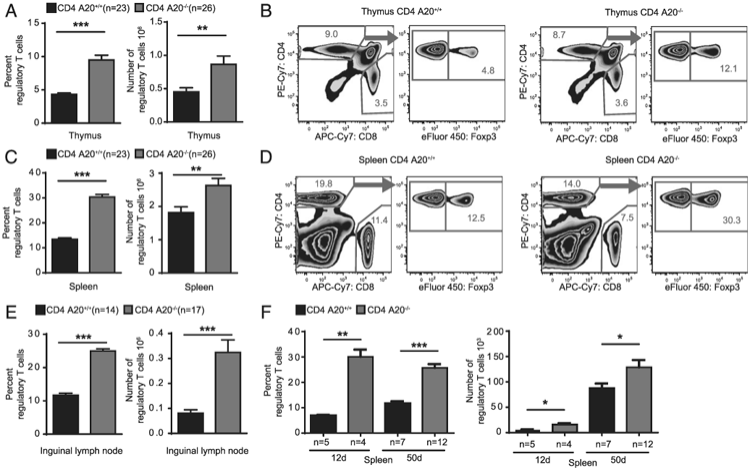
Increased numbers of T_reg_ cells in mice lacking A20 specifically in T cells. (**A**) Thymocytes of adult A20^F/F^ CD4^Cre−^ (CD4 A20^+/+^) and A20^F/F^ CD4^Cre+^ (CD4 A20^−/−^) mice were stained with anti-CD4, anti-CD8, anti-Foxp3, and live/dead reagent. The population of Foxp3^+^ T_reg_ cells of live CD4^+^ CD8^−^ cells was determined by flow cytometry (left panel), and total T_reg_ cell number was calculated (right panel). Pooled data of five independent experiments are shown. Animal numbers per group (*n*) are depicted. (**B**) Gating strategy and representative FACS plots of the experiments depicted in (A). (**C**) Splenocytes of adult CD4 A20^+/+^ and CD4 A20^−/−^ mice were stained as in (A). The population of Foxp3^+^ T_reg_ cells of live CD4^+^ cells was determined by flow cytometry (left panel), and total T_reg_ cell number was calculated (right panel). Pooled data of five independent experiments are shown. Animal numbers per group (*n*) are depicted. (**D**) Gating strategy and representative FACS plots of the experiments depicted in (C). (**E**) Lymph node cells of adult CD4 A20^+/+^ and CD4 A20^−/−^ mice were stained as in (A). The Foxp3^+^ T_reg_ cell fraction of all live CD4^+^ cells was determined by flow cytometry. Pooled data of three independent experiments are shown. Animal numbers per group (*n*) are depicted. (**F**) Splenocytes of 12- and 50-d–old CD4 A20^+/+^ and CD4 A20^−/−^ mice were analyzed for CD4 and Foxp3 expression by flow cytometry. T_reg_ cell fractions of live CD4 cells and absolute T_reg_ cell numbers are shown. Pooled data of three independent experiments are shown. Animal numbers per group (*n*) are depicted. Data were analyzed using ordinary one-way ANOVA for multiple comparisons or one- and two-tailed unpaired *t* test. Data are presented as mean ± SEM. Significance was set at *p < 0.05, **p < 0.01, ***p < 0.001.

To investigate the role of A20 in T cells, we analyzed A20^F/F^ CD4^Cre+^ mice, which specifically lack A20 in T cells. The population of Foxp3^+^ T_reg_ cells was quantitatively enlarged in thymus, spleen, and inguinal lymph nodes of naive A20^F/F^ CD4^Cre+^ mice (CD4 A20^−/−^) as compared with A20^F/F^ CD4^Cre−^ mice (CD4 A20^+/+^) (Fig. 1A–E, Supplemental Fig. 1A). At the same time, the total thymic CD4^+^ and CD8^+^ T cell population was not significantly different between CD4 A20^−/−^ mice and controls. In the spleen, both total CD4^+^ and CD8^+^ T cell populations were reduced in CD4 A20^−/−^ mice (Fig. 1B, 1D, Supplemental Fig. 1B, 1C), which is in line with previous observations (38). To exclude a bias of age on the development of T_reg_ cells (43), we analyzed splenocytes of young (12-d-old) and adult (50-d-old) CD4 A20^+/+^ and CD4 A20^−/−^ mice. We found frequencies and absolute numbers of T_reg_ cells to be significantly increased in both young and adult mice (Fig. 1F).

### A20-deficient T cells show reduced T_reg_ cell expansion and differentiation in vitro

**Figure 2:**
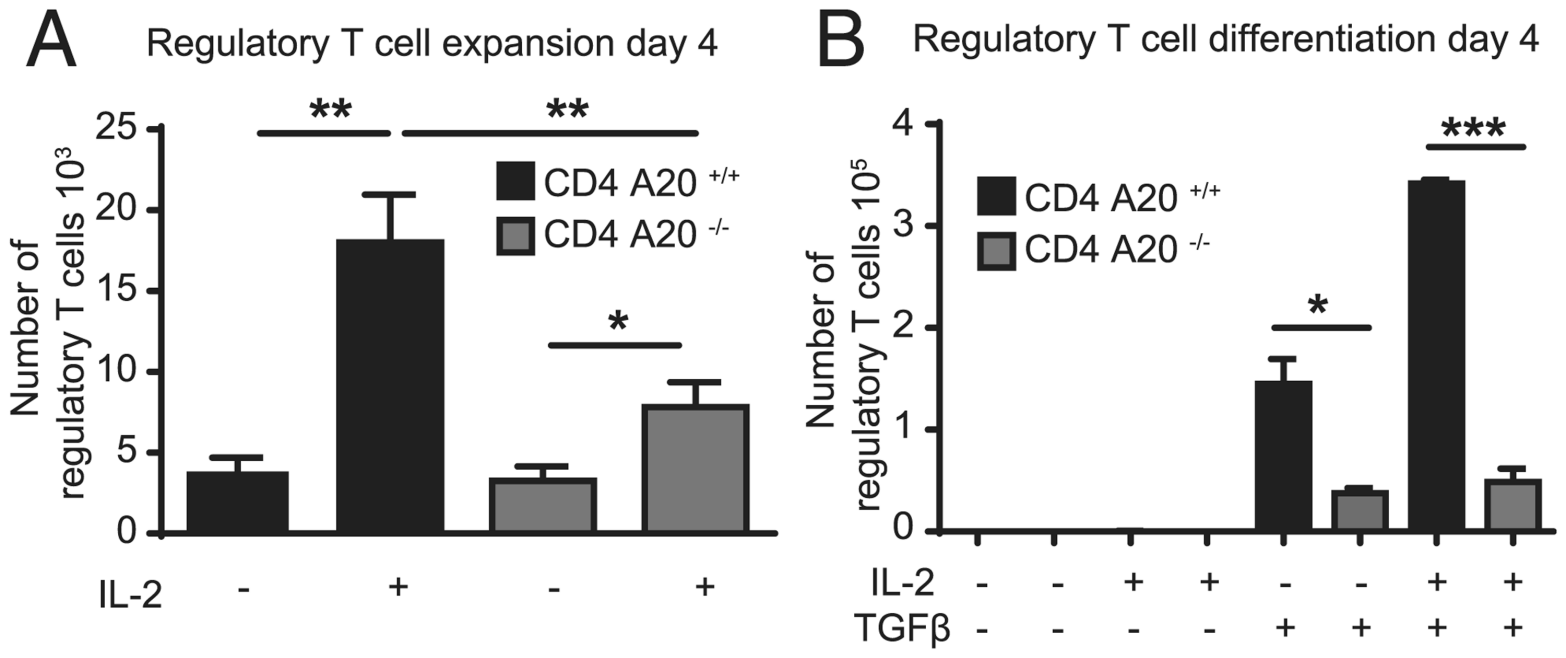
A20-deficient T cells show reduced T_reg_ cell expansion and differentiation in vitro. (**A**) A total of 50 × 10^3^ FACS-sorted A20^+/+^ and A20^−/−^ CD4^+^ CD25^+^ T_reg_ cells was cultured in vitro in the presence of plate-bound anti-CD3 and soluble anti-CD28 with or without IL-2 for 4 d, and absolute numbers of live cells were determined. One representative of three independent experiments is shown. (**B**) A total of 40 × 10^3^ CD4^+^ CD62L^high^ CD44^low^ A20^+/+^ and A20^−/−^ T cells was cultured in vitro in the presence of plate-bound anti-CD3 and soluble anti-CD28, ± IL-2 and ± TGF-β for 4 d. Conversion toward a T_reg_ cell phenotype was determined by intracellular Foxp3 staining, and absolute numbers of live cells were determined. One representative of three independent experiments is shown. Data were analyzed using ordinary one-way ANOVA for multiple comparisons and two-tailed unpaired *t* test. Data are presented as mean ± SEM. Significance was set at *p < 0.05, **p < 0.01, ***p < 0.001.

To investigate whether this absolute increase of peripheral T_reg_ cell numbers in CD4 A20^−/−^ mice was due to enhanced sensitivity of T_reg_ cells to external proliferation signals, we stimulated FACS-purified CD4^+^ CD25^+^ T_reg_ cells from CD4 A20^+/+^ and CD4 A20^−/−^ mice in vitro with anti-CD3, anti-CD28, and IL-2 (44). Although IL-2 induced a significant increase in the absolute numbers of both control and A20-deficient T_reg_ cells after 4 or 8 d of culture, the in vitro expansion of A20-deficient T_reg_ cells was significantly less pronounced (Fig. 2A, Supplemental Fig. 2A). Next, we wanted to know whether enhanced sensitivity to peripheral differentiation signals of T_reg_ cells was responsible for the enlarged T_reg_ cell population in CD4 A20^−/−^ mice. However, stimulation of FACS-purified CD62L^high^ CD44^low^ naive T cells with anti-CD3, anti-CD28, and either TGF-β alone or TGF-β and IL-2 resulted in decreased proportions and absolute numbers of A20-deficient compared with control T_reg_ cells (Fig. 2B, Supplemental Fig. 2B). In summary, the sensitivity to expansion or differentiation signals of A20-deficient T_reg_ cells or CD4^+^ T cells in vitro was reduced compared with controls, and thus could not explain the increase of total T_reg_ cell numbers in CD4 A20^−/−^ mice.

### A20 deficiency in T cells drives thymic T_reg_ cell development cell-intrinsically

**Figure 3:**
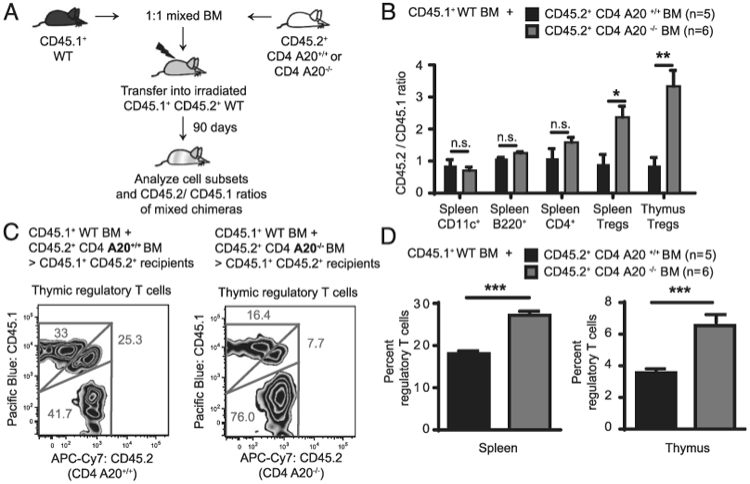
A20 deficiency in T cells drives thymic T_reg_ cell development cell-intrinsically. (**A**) CD45.1^+^ CD45.2^+^ double-positive C57BL/6 recipient mice received 11 Gy TBI and were then transplanted with 2.5 × 10^6^ T cell–depleted C57BL/6 WT BM expressing only CD45.1. In addition, recipient mice received either 2.5 × 10^6^ CD4 A20^+/+^ or 2.5 × 10^6^ CD4 A20^−/−^ T cell–depleted C57BL/6 BM, both expressing only CD45.2. (**B**) Three months after transplantation, numbers and frequencies of CD11c^+^ dendritic cells, B220^+^ B cells, CD4^+^ T cells, and CD4^+^ CD8^−^ Foxp3^+^ T_reg_ cells were determined in thymus and spleen of corresponding animals by FACS analysis. CD45.1 and CD45.2 expression of cell subsets were analyzed, and CD45.2^+^/CD45.1^+^ ratios were calculated. Residual recipient cells were identified (CD45.1^+^ CD45.2^+^ double-positives) and were excluded from the calculation. One representative of two independent experiments is shown. Animal numbers per group (*n*) are depicted. (**C**) Gating strategy and representative FACS plots of thymic T_reg_ cells of the analysis depicted in (B). (**D**) Percentage of Foxp3^+^ T_reg_ cells of CD4^+^ CD8^−^ cells were determined in thymus and spleen of recipient mice that were transplanted as described in (A). One representative of two independent experiments is shown. Animal numbers per group (*n*) are depicted. Experiments were analyzed using two-tailed unpaired *t* test. Data are presented as mean ± SEM. Significance was set at *p < 0.05, **p < 0.01, ***p < 0.001.

We assumed that an intrinsic effect could account for increased T_reg_ cell development and enhanced T_reg_ cell numbers in CD4 A20^−/−^ animals. To test this hypothesis, we established a model of competitive BM engraftment and T cell development in which CD45.1^+^ CD45.2^+^ double-positive C57BL/6 recipient mice were lethally irradiated (TBI) to eradicate the recipient hematopoietic system. Then T cell–depleted BM cells mixed in a 1:1 ratio from both CD45.1 (WT) and CD45.2 (either CD4 A20^+/+^ or CD4 A20^−/−^) donors (Fig. 3A) were introduced for hematopoietic reconstitution.

In this model, tracing the genetic markers by FACS analysis allows to quantify whether one of the BM compartments transferred to the host at day 0 (WT plus CD4 A20^+/+^ or WT plus CD4 A20^−/−^) would outperform another or would favor the development of certain T cell or other immune cell subsets. Ninety days after transplantation, recipients that had received CD45.1 WT + CD45.2 CD4 A20^+/+^ BM showed CD45.2 and CD45.1 T_reg_ cell fractions with a balanced ratio. In contrast, recipients that had received CD45.1 WT + CD45.2 CD4 A20^−/−^ BM showed an ∼2.6-fold (spleen) and 3.8-fold (thymus) increased ratio between CD45.2^+^ A20^−/−^ and CD45.1 WT T_reg_ cell fractions (Fig. 3B, 3C). Ratios of CD45.2/CD45.1 populations of splenic dendritic cells, B cells, and total CD4^+^ T cells did not differ significantly between CD4 A20^+/+^ BM or CD4 A20^−/−^ BM (Fig. 3B). Accordingly, we found increased fractions of Foxp3^+^ T_reg_ cells within the CD4^+^ T cell compartment in spleen and thymus of mice that had received CD45.1 WT plus CD4 A20^−/−^ BM compared with mice that had received CD45.1 WT plus CD4 A20^+/+^ BM (Fig. 3D), whereas populations of CD11c^+^, B220^+^, and CD4^+^ T cells were similar in both cohorts (Supplemental Fig. 2C). Together with our findings that in vitro T_reg_ cell differentiation and expansion through common external stimuli were not enhanced in CD4 A20^−/−^ cells, these data suggest that early events in the BM or thymus favor T_reg_ cell development in CD4 A20^−/−^ mice via cell-intrinsic mechanisms.

### A20 deficiency reduces the dependence of thymic T_reg_ cells on IL-2

**Figure 4:**
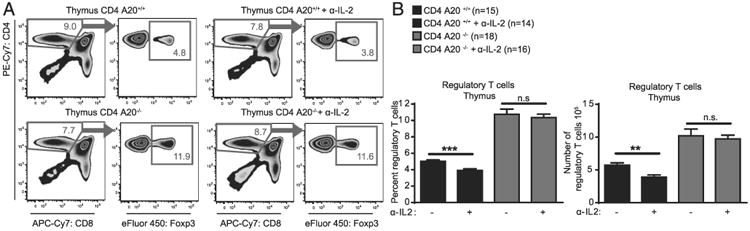
A20 deficiency reduces the dependence of thymic T regulatory cells on IL-2. (**A**) CD4 A20^+/+^ or CD4 A20^−/−^ mice were injected i.p. with anti–IL-2 neutralizing Ab and analyzed 6 d after first treatment. One group of each genotype was left without anti–IL-2 treatment as control. Thymocytes were stained with anti-CD4, anti-CD8, anti-Foxp3, and live/dead reagent, and the population of Foxp3^+^ T_reg_ cells of live CD4^+^ CD8^−^ cells was determined by flow cytometry. Shown are the gating strategy and representative FACS plots of thymic T_reg_ cells. (**B**) Experiments and analyses as described in (A). (Left panel) Frequency of Foxp3^+^ T_reg_ cells of CD4^+^ CD8^−^ cells. (Right panel) Absolute number of Foxp3^+^ CD4^+^ CD8^−^ T_reg_ cells. Pooled data of three independent experiments are shown. Animal numbers per group (*n*) are depicted. Experiments were analyzed using two-tailed unpaired *t* test. Data are presented as mean ± SEM. Significance was set at **p < 0.01, and ***p < 0.001.

IL-2 was shown to play a crucial role in thymic T_reg_ cell development by stabilizing the Foxp3^+^ phenotype and counterbalancing Foxp3-induced apoptosis (45). To investigate effects of IL-2 signaling on A20^−/−^ T_reg_ cells, we used in vivo application of an anti–IL-2 Ab that antagonizes effects of IL-2 (46). We injected anti–IL-2 into naive CD4 A20^+/+^ and CD4 A20^−/−^ mice, and analyzed T cell frequencies and numbers 6 d later. The number of total thymocytes and the frequency of CD4 single-positive (CD4SP) T cells were unchanged after 6 d in all groups (Fig. 4A, Supplemental Fig. 2D). However, frequencies and numbers of T_reg_ cells in the thymus of CD4 A20^+/+^ mice were significantly reduced 6 d after injection of anti–IL-2 (Fig. 4). In contrast, CD4 A20^−/−^ mice showed unchanged T_reg_ frequencies after neutralization of IL-2 (Fig. 4). We examined the expression of the integral IL-2R component IL-2RA (CD25) in the population of Foxp3^+^ CD4^+^ T_reg_ cells to test whether differences in IL-2R expression, which could possibly lead to altered sensitivity for IL-2, contributed to this phenotype. We found similar proportions of CD25^+^ and CD25^−^ Foxp3^+^ T_reg_ cells in CD4 A20^+/+^ or CD4 A20^−/−^ mice and marginally decreased CD25 expression levels of CD25^+^ Foxp3^+^ T_reg_ cells in CD4 A20^−/−^ mice (Supplemental Fig. 2E). Overall, these data indicate that the dependence of thymic T_reg_ cell development on IL-2 is reduced in the absence of A20.

### Thymic A20-deficient T_reg_ cells show unchanged proliferation and apoptosis

**Figure 5:**
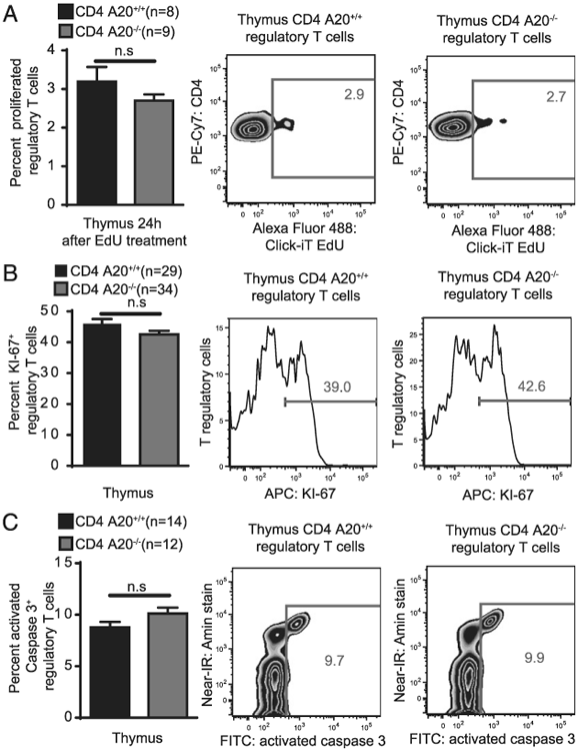
Thymic A20-deficient T_reg_ cells show unchanged proliferation and apoptosis. (**A**) CD4 A20^+/+^ and CD4 A20^−/−^ mice were treated with 50 mg/kg EdU. Twenty-four hours after application, harvested thymocytes were stained with live/dead reagent, anti-CD4, anti-CD8, and anti-Foxp3, and EdU Click-iT staining was performed. The population of EdU^+^ T_reg_ cells of all Foxp3^+^ T_reg_ cells was determined by flow cytometry. Pooled data of two independent experiments are shown (left panel). Animal numbers per group (*n*) are depicted. Gating strategy and representative FACS plots of the experiments are shown (right panels). (**B**) Thymocytes of adult CD4 A20^+/+^ and CD4 A20^−/−^ mice were stained with live/dead reagent, anti-CD4, anti-CD8, anti-Foxp3, and anti–KI-67. The population of KI-67^+^ T_reg_ cells of all Foxp3^+^ T_reg_ cells was determined by flow cytometry. Pooled data of six independent experiments are shown (left panel). Animal numbers per group (*n*) are depicted. Gating strategy and representative histograms of the experiments are shown (right panels). (**C**) Thymocytes of adult CD4 A20^+/+^ and CD4 A20^−/−^ mice were incubated with an active caspase-3 staining reagent for 45 min and stained with live/dead reagent, anti-CD4, anti-CD8, and anti-Foxp3. The population of activated caspase-3^+^ T_reg_ cells of all Foxp3^+^ T_reg_ cells was determined by flow cytometry. Pooled data of three independent experiments are shown (left panel). Animal numbers per group (*n*) are depicted. Gating strategy and representative FACS plots of the experiments are shown (right panels). Experiments were analyzed using two-tailed unpaired *t* test. Data are presented as mean ± SEM. Significance was set at *p* < 0.05.

We next wanted to analyze the role of A20 for T_reg_ proliferation and apoptosis in vivo. First, we treated CD4 A20^−/−^ and CD4 A20^+/+^ mice with EdU, a nucleoside analog that is incorporated into DNA during active DNA synthesis and can be analyzed by flow cytometry. We found similar frequencies of EdU^+^ thymic T_reg_ cells in CD4 A20^−/−^ and CD4 A20^+/+^ mice 24 h after EdU injection (Fig. 5A). We then tested the expression rate of the proliferation marker KI-67 (47) in thymic T_reg_ cells and again found no significant difference between CD4 A20^−/−^ and CD4 A20^+/+^ mice (Fig. 5B). Next, we determined the frequency of thymic T_reg_ cells that stain positive for activated caspase-3 to evaluate the role of apoptosis in T_reg_ cells but found no significant difference between CD4 A20^−/−^ and CD4 A20^+/+^ mice (Fig. 5C). In contrast, when analyzing Foxp3^−^, CD4SP thymic cells, we observed reduced frequencies of EdU^+^, similar frequencies of KI-67^+^, and increased frequencies of activated caspase-3^+^ cells in CD4 A20^−/−^ versus A20^+/+^ mice (Supplemental Fig. 3). Our data indicate that A20 does not regulate T_reg_ cell proliferation or apoptosis in vivo under the conditions tested in our study and suggest that these mechanisms are not responsible for the enlarged T_reg_ cell population in CD4 A20^−/−^ mice.

### Thymic A20-deficient T_reg_ cells show enhanced RelA phosphorylation and reduced nuclear translocation of c-Rel

**Figure 6:**
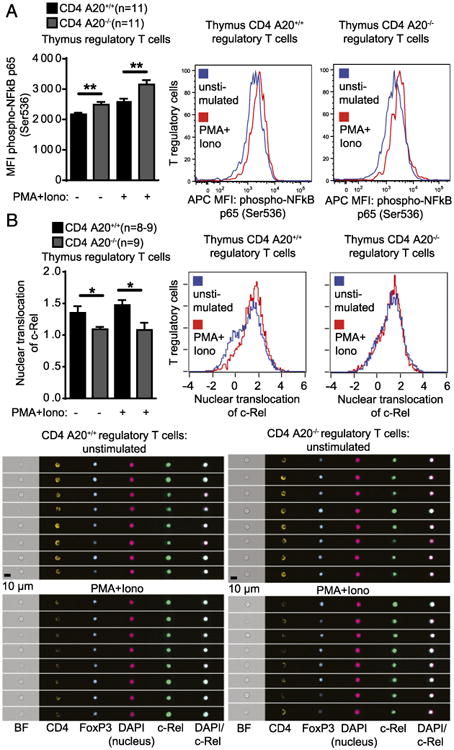
Thymic A20-deficient T_reg_ cells show enhanced RelA activation and reduced nuclear trans-location of c-Rel. (**A**) Thymocytes of adult CD4 A20^+/+^ and CD4 A20^−/−^ mice were left unstimulated or stimulated with PMA and ionomycin (Iono) for 30 min and stained with live/dead reagent, anti-CD4, anti-CD8, anti-Foxp3, and anti–phospho-NF-κB p65. Median fluorescence intensity (MFI) of phospho–NF-κB of CD4^+^ CD8^−^ (CD4SP) Foxp3^+^ T_reg_ cells was determined by flow cytometry. Pooled data of two independent experiments are shown. Animal numbers per group (*n*) are depicted (left panel). Representative histograms showing fluorescence intensity of phospho–NF-κB p65 of T_reg_ cells are shown (right panels). (**B**) CD8^+^ cell MACS-depleted thymocytes of CD4 A20^+/+^ and CD4 A20^−/−^ mice were left unstimulated or stimulated with PMA and Iono for 30 min; stained with anti-CD4, anti-CD8, anti-Foxp3, anti–c-Rel, and DAPI; and acquired on an imaging flow cytometer. Nuclear translocation of CD4SP Foxp3^+^ T_reg_ cells was quantified based on the similarity score of c-Rel and nuclear image intensities. Pooled data of two independent experiments are shown. Animal numbers per group (*n*) are depicted (left panel). Representative histograms show the nuclear translocation score of unstimulated and stimulated CD4SP Foxp3^+^ T_reg_ cell populations (right panels). Exemplary images are representative for the mean nuclear translocation score of indicated populations (lower panels). Experiments were analyzed using two-tailed unpaired *t* test. Data are presented as mean ± SEM. Significance was set at *p < 0.05 and **p < 0.01.

Because A20 was shown to be a central negative regulator of NF-κB activity in several cell types, we then asked whether deleting A20 in CD4^+^ T cells led to increased NF-κB activity in those cells. In fact, both Foxp3^+^ CD4SP thymic T_reg_ cells and Foxp3^−^ CD4SP thymic T cells displayed increased NF-κB activity as measured by p65 phosphorylation intensity after stimulation. In Foxp3^+^ CD4SP thymic T_reg_ cells, p65 phosphorylation intensity was already increased at baseline without stimulation, which was also the case for Foxp3^−^ CD4SP thymic T cells (Fig. 6A, Supplemental Fig. 4A). Because the NF-κB transcription factor c-Rel was shown to play a pronounced role in thymic T_reg_ cell development (17), we assessed c-Rel translocation to the nucleus of thymic T_reg_ cells on single-cell levels. Although c-Rel translocation was somewhat decreased in thymic Foxp3^+^ CD4SP A20^−/−^ T_reg_ cells compared with A20^+/+^ T_reg_ cells, thymic T_reg_ cells were characterized by a high degree of nuclear c-Rel translocation in both genotypes even in the absence of stimulation (Fig. 6B). Foxp3^−^ CD4SP thymic A20^−/−^ T cells also showed a trend for reduced nuclear c-Rel translocation (Supplemental Fig. 4B).

### A20 limits development of thymic T_reg_ cell progenitors

**Figure 7:**
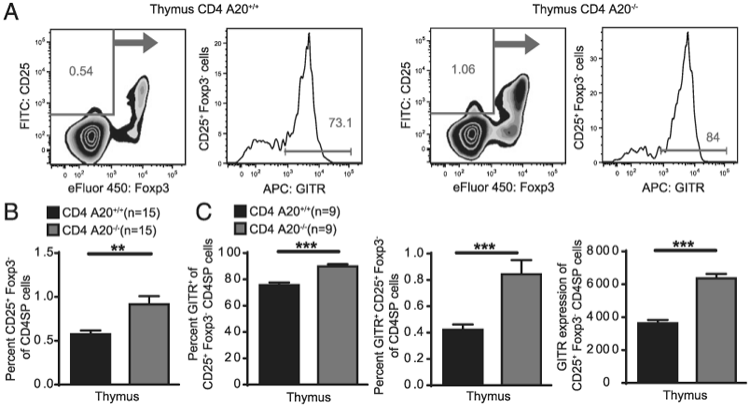
A20 limits development of thymic T_reg_ cell progenitors. (**A**) Thymocytes of adult CD4 A20^+/+^ and CD4 A20^−/−^ mice were stained with anti-CD4, anti-CD8, anti-CD25, anti-Foxp3, anti-GITR, and live/dead reagent. The population of CD4^+^ CD8^−^ CD25^+^ Foxp3^−^ GITR^+^ live cells was determined by flow cytometry. Gating strategy and representative FACS plots of cells that were previously gated on single, live CD4^+^ CD8^−^ (CD4SP) thymocytes are depicted. (**B**) Frequencies of CD25^+^ Foxp3^−^ of CD4SP live cells were determined by flow cytometry. Pooled data of three independent experiments are shown. Animal numbers per group (*n*) are depicted. (**C**) Frequencies of GITR^+^ cells of CD25^+^ Foxp3^−^ CD4SP cells (left panel), frequencies of GITR^+^ CD25^+^ Foxp3^−^ of CD4SP live cells (middle panel), and mean GITR expression of CD25^+^ Foxp3^−^ CD4SP live cells (right panel) were determined by flow cytometry. Pooled data of two independent experiments are shown. Animal numbers per group (*n*) are depicted. Experiments were analyzed using two-tailed unpaired *t* test. Data are presented as mean ± SEM. Significance was set at **p < 0.01 and ***p < 0.001.

Considering the observations that NF-κB transcription factors and especially c-Rel modulates various steps of T_reg_ cell development including the generation of thymic T_reg_ cell progenitors before Foxp3 expression (48), we examined thymic T_reg_ precursors in the absence of A20. Mice with A20^−/−^ T cells exhibited increased frequencies of CD25^+^ Foxp3^−^ CD4SP thymocytes, revealing an enhanced T_reg_ cell precursor compartment (Fig. 7A, 7B). Furthermore, we observed an increased fraction of GITR^+^ cells within this population, resulting in a ∼2-fold increased T_reg_ cell progenitor (CD4SP CD25^+^ GITR^+^ Foxp3^−^) compartment (Fig. 7C). In addition, GITR expression of the CD25^+^ Foxp3^−^ CD4SP A20^−/−^ thymic T_reg_ precursors was enhanced compared with Foxp3-expressing T_reg_ cells (Fig. 7C, Supplemental Fig. 4C). This population increase at the T_reg_ precursor differentiation stage in CD4 A20^−/−^ mice suggests that early intrinsic mechanisms preceding Foxp3 expression are responsible for the developmental advantage of the CD4 A20^−/−^ versus CD4 A20^+/+^ T_reg_ cell population.

### A20-deficient T_reg_ cells are functional

**Figure 8:**
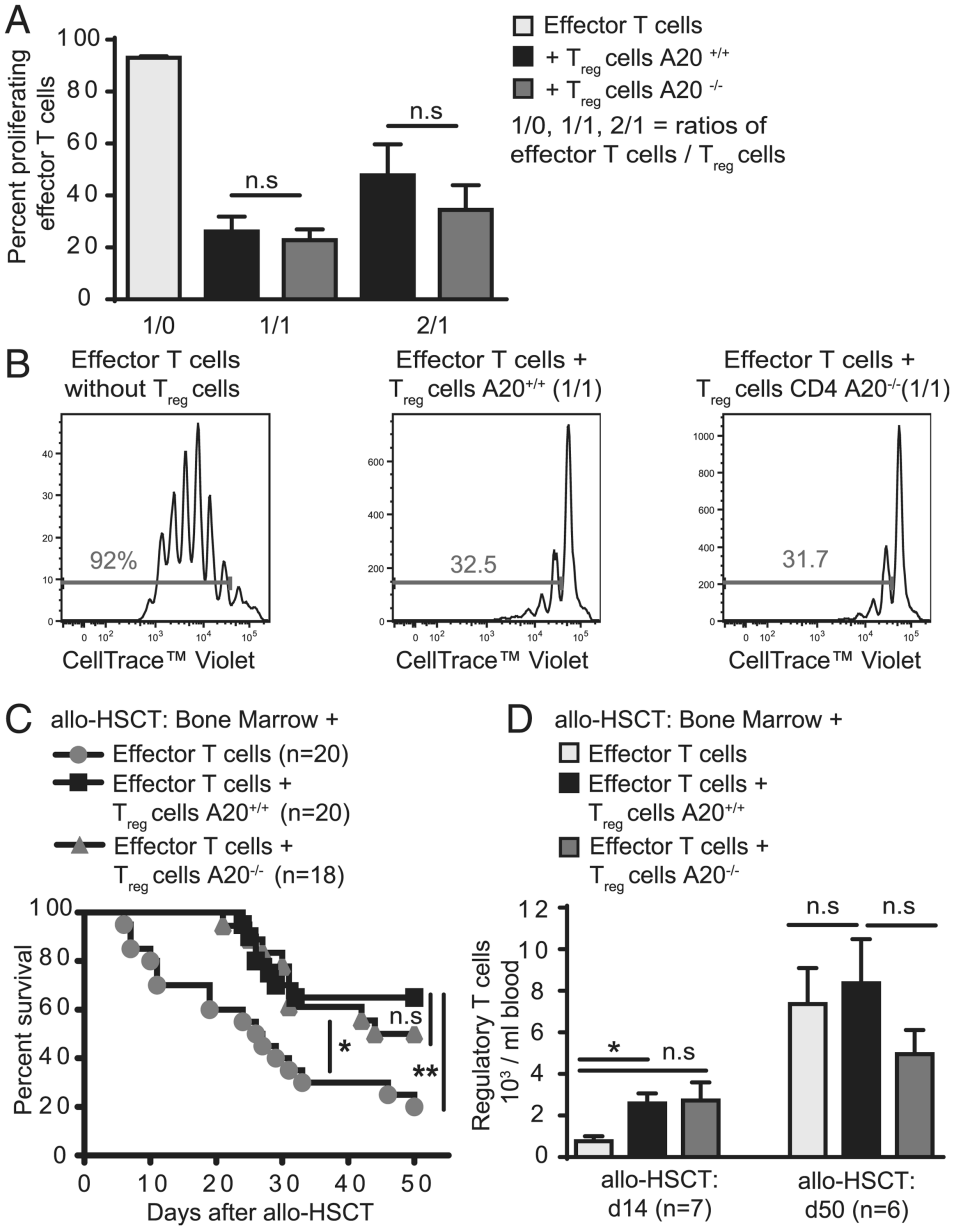
A20-deficient T_reg_ cells are functional. (**A**) MACS-sorted A20^+/+^ or A20^−/−^ CD4^+^ CD25^−^ T effector cells were labeled with a cell tracer dye (CellTrace Violet) and cultured together with CD4^+^ CD25^+^ T_reg_ cells in the presence of plate-bound anti-CD3 and irradiated APCs at different ratios. Numbers of proliferating effector T (T_eff_) cells were determined on day 4. Pooled data of two independent experiments are shown. (**B**) Gating strategy and representative histograms of labeled T_eff_ cells of one representative experiment described in (A). (**C**) Survival curve of allo-HSCT recipient mice (BALB/c) after 9 Gy TBI + 5 × 10^6^ T cell–depleted BM cells + 2 × 10^5^ CD4^+^ CD25^−^ + 2 × 10^5^ CD8^+^ T cells (C57BL/6) and either 6 × 10^5^ A20^+/+^ or A20^−/−^ CD4^+^ CD25^+^ T_reg_ cells (C57BL/6). Pooled data of three independent experiments are shown. Animal numbers per group (*n*) are depicted. (**D**) Number of donor-derived T_reg_ cells in the blood of allo-HSCT recipient mice that were transplanted as described in (C). Blood was taken at indicated time points. The experiment was performed once. Animal numbers per group (*n*) are depicted. Survival was analyzed using the log-rank test. Other experiments were analyzed using two-tailed unpaired *t* test. Data are presented as mean ± SEM. Significance was set at *p < 0.05 and **p < 0.01.

Having demonstrated that deleting A20 in CD4 T cells induces a specific T_reg_ cell population increase, we next analyzed whether A20-deficient T_reg_ cells are functional. In vitro, we found that CD4^+^ CD25^+^ T_reg_ cells purified from CD4 A20^+/+^ or CD4 A20^−/−^ mice both efficiently suppressed proliferation of A20^+/+^ T effector cells to a similar extent (Fig. 8A, 8B). To analyze T_reg_ cell function in vivo, we used a model of major mismatch allogeneic hematopoietic stem cell transplantation (allo-HSCT), in which the hematopoietic system of recipient mice is eradicated by TBI and then reconstituted with BM and T cells from allogeneic donors. Lethal effects of acute GVHD, mediated by allogeneic effector T cells, can be significantly reduced in this model by cotransplantation of T_reg_ cells (49). Survival of mice that received either A20^+/+^ or A20^−/−^ T_reg_ cells together with WT donor BM and T cells was similarly increased in both groups compared with controls that did not receive additional T_reg_ cells (Fig. 8C). This was attributable to the function of individual T_reg_ cells and not due to numerical changes throughout the course of the experiment, because the absolute number of T_reg_ cells in animals cotransplanted with effector and T_reg_ cells was not significantly different when CD4 A20^+/+^ or CD4 A20^−/−^ T_reg_ cells were used (Fig. 8D). Together, our data show that loss of A20 does not impair the suppressive functions of T_reg_ cells.

## Discussion

NF-κB activation modulates T_reg_ cell development through various mechanisms, including induction of Foxp3 transcription in the thymus (16). We hypothesized that enhanced NF-κB activity in CD4^+^ T cells, as in the absence of its negative regulator A20, might have an impact on the development, maintenance, and functionality of T_reg_ cells. We observed that mice lacking A20 specifically in CD4^+^ T cells had increased numbers of CD4^+^ T_reg_ cells in spleen, lymph nodes, and thymus.

Because the increase in T_reg_ cells, which are known to arise in the thymus very early after birth (50), was already detectable in 12-d-old mice and could not be explained by enhanced iT_reg_ cell differentiation or expansion, we hypothesized that a cell-intrinsic effect during thymic T_reg_ cell generation may account for T_reg_ cell expansion in CD4 A20^−/−^ mice. In fact, competitive engraftment experiments in chimeras with BM from CD4 A20^−/−^ mice equally mixed with BM from WT mice established a significantly larger T_reg_ cell compartment compared with chimeras with BM from CD4 A20^+/+^ mice equally mixed with BM from additional WT mice. We observed that the majority of T_reg_ cells contributing to the enlarged T_reg_ cell compartment were derived from A20^−/−^ BM. This is in line with previous findings that increased NF-κB activity, in particular of c-Rel (17), leads to increased proportions and absolute numbers of thymic Foxp3^+^ natural T_reg_ cells (16).

Complementary to our data with CD4 A20^−/−^ BM chimeras, less T_reg_ cells were generated from c-Rel–deficient BM than from WT BM in a competitive setting (18). The differentiation of naive T cells toward iT_reg_ cells was regulated by c-Rel independently of IL-2 (18). Remarkably, in our setting, CD4 A20^−/−^ T_reg_ cells were less responsive to IL-2 in vitro. IL-2 is known to maintain T_reg_ cells by preventing Foxp3-induced apoptosis (46) and through regulation of MCL-1 that is essential for T_reg_ cell survival (45). In this context, we observed that A20^−/−^ T_reg_ cells were less affected by both the presence and absence of IL-2. Although the T_reg_ compartment originating from CD4 A20^−/−^ BM was expanded in vivo, A20^−/−^ cells displayed a reduced response to classical T_reg_ cell proliferative or differentiating factors such as IL-2 and TGF-β in vitro. In vivo, neither proliferation nor apoptosis was significantly affected in A20-deficient T_reg_ cells, contrary to A20^−/−^ effector T cells, which showed increased rates of apoptosis. This is in line with previous findings that show that A20 regulates cell death and survival of effector T cells (34, 35, 38). At the current stage, the molecular mechanisms to explain the relative insensitivity of A20^−/−^ T_reg_ cells toward IL-2 remain unclear. Intrinsic effects of differential NF-κB regulation in CD4 A20^−/−^ mice rather than a higher cell turnover are the most likely explanation for T_reg_ expansion in these mice in vivo. In this context, we found that the activity of the NF-κB subunit RelA (p65) was enhanced in Foxp3^+^ thymic T_reg_ cells in the absence of A20. In contrast, A20^−/−^ thymic T_reg_ cells showed reduced nuclear translocation of the NF-κB subunit c-Rel. Because NF-κB signaling was shown to modulate multiple steps of T_reg_ cell development in the thymus (48), we hypothesize that A20 influences the thymic development of natural T_reg_ cells through modulation of NF-κB activation in T_reg_ cell progenitors. In line with this hypothesis, we discovered that the population increase in CD4 A20^−/−^ compared with CD4 A20^+/+^ mice was not restricted to the Foxp3^+^ thymic T_reg_ cell compartment, but was also comparably elevated in CD4SP CD25^+^ GITR^+^ Foxp3^−^ thymic T_reg_ cell progenitors. Furthermore, we observed elevated GITR expression levels of CD4SP CD25^+^ Foxp3^−^ thymic T_reg_ cell precursors. In this regard, it was shown recently that costimulation of tumor necrosis factor receptor superfamily molecules, such as GITR, connects TCR signal strength to thymic differentiation of T_reg_ cells (51). Further studies are needed to examine a possible role of A20 and NF-κB activation in this context and to define how A20 restrains thymic T_reg_ cell progenitor generation molecularly. In addition, whether deleting A20 at other time points of T_reg_ cell generation that coincide with CD4 expression would entail different outcomes remains to be determined.

Our finding that A20 restricts thymic T_reg_ cell generation is consistent with previous data for CYLD, another negative regulator of NF-κB, which, as A20, was shown to remove nonproteolytic K63-linked polyubiquitin chains from signaling molecules in the TCR pathway. CYLD deficiency promotes constitutive NF-κB activity in thymocytes and peripheral T cells, and is associated with enhanced T_reg_ cell frequencies (52, 53). Interestingly, CD25 expression is decreased in CYLD-deficient T_reg_ cells (52), which is in line with our findings revealing lower expression levels of CD25 in A20^−/−^ CD4^+^ Foxp3^+^ CD25^+^ T_reg_ cells. However, CYLD-deficient T_reg_ cells were shown to be less functional, failing to inhibit colitis in an adoptive transfer colitis model (52). This is in contrast with our data demonstrating that increased natural A20^−/−^ T_reg_ cells are functional to suppress allogeneic effector T cell toxicity in a preclinical model of acute GVHD. Perhaps differential NF-κB regulation may explain2 those discrepancies, because A20 terminates NF-κB signaling (19), whereas CYLD prevents spontaneous NF-κB activation (54).

In summary, we propose that A20 holds the thymic development of natural T_reg_ cells in check and thereby contributes to fine-tuning the CD4^+^ T cell response. Given that A20 inhibits T effector cell death in CD4^+^ T cells, unleashing T effector cell–mediated inflammatory activity, our data complement the picture of A20 as a potent regulator of T cell–mediated inflammation, inducing T effector cell survival while reducing T_reg_ cell generation. In light of the largely anti-inflammatory effects that have been attributed to A20 in many cell types, this proinflammatory aspect of A20 physiology in effector and regulatory CD4^+^ T cells is particularly important because it may contribute to a change of perception of the functions of A20 as a negative regulator of NF-κB in the context of inflammation. Whether targeted modulation of A20 activity may allow to promote T_reg_ cell–mediated immune tolerance or, alternatively, favorable T cell immunity is a question of translational relevance that needs to be addressed in the future.

## Supplementary Material

supplemental

## Abbreviations used in this article

allo-HSCT: allogeneic hematopoietic stem cell transplantation
BM: bone marrow
γc: common γ-chain
CD4SP: CD4 single-positive
EdU: 5-ethynyl-2′-deoxyuridine
GVHD: graft-versus-host disease
iT_reg_: induced T_reg_
TBI: total body irradiation
T_reg_: regulatory T
WT: wild type
