# Supplementary material for: A20 Restrains Thymic Regulatory T Cell Development
Source: J Immunol. Author manuscript; Available in PMC 2018 Apr 1. (PMC5617121; doi:10.4049/jimmunol.1602102)
Supplement: supplemental [file NIHMS906294-supplement-supplemental.pdf]

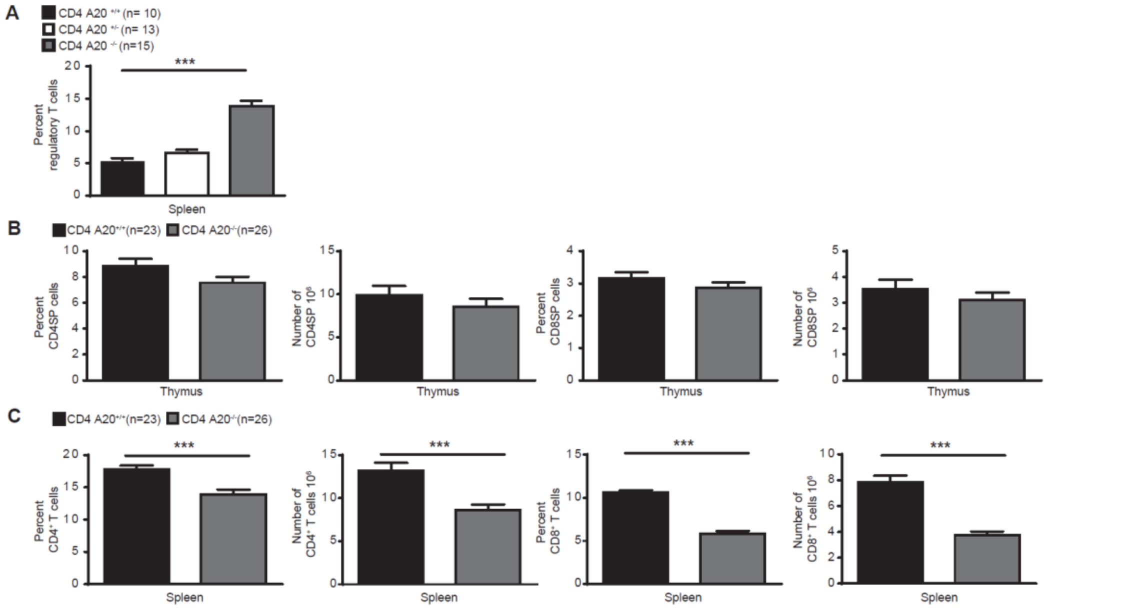

Supplementary Figure 1

Supplementary Figure 1: A20-deficient T cells show a reduced CD4$^+$ and CD8$^+$ splenic T cell compartment

A) Splenocytes of adult A20$^{F/F}$ CD4^{Cre-} (CD4 A20$^{+/+}$), A20$^{F/+}$ CD4^{Cre+} (CD4 A20$^{+/-}$) and A20$^{F/F}$ CD4^{Cre+} (CD4 A20$^{-/-}$) mice were stained with anti-CD4, anti-Foxp3 and live/dead reagent. The population of Foxp3$^+$ T$_{reg}$ cells of live CD4$^+$ cells was determined by flow cytometry. Pooled data of 3 independent experiments are shown. Animal numbers per group (n) are depicted. **B)** Thymocytes of adult CD4 A20$^{+/+}$ and CD4 A20$^{-/-}$ mice were stained with anti-CD4, anti-CD8, anti-Foxp3 and live/dead reagent. The population of CD4$^+$ CD8$^-$ (CD4SP) or CD4$^-$ CD8$^+$ (CD8SP) cells of all live cells was determined by flow cytometry and total CD4SP or CD8SP cell number was calculated. Pooled data of 5 independent experiments are shown. Animal numbers per group (n) are depicted. **C)** Splenocytes of adult CD4 A20$^{+/+}$ and CD4 A20$^{-/-}$ mice were analyzed as described in B. Pooled data of 5 independent experiments are shown. Animal numbers per group (n) are depicted. Data were analyzed using two-tailed unpaired t test. Significance was set at $p < 0.05$, $p < 0.01$ and $p < 0.001$ and was then indicated with asterisks (*, ** and ***). Data are presented as mean ± S.E.M.

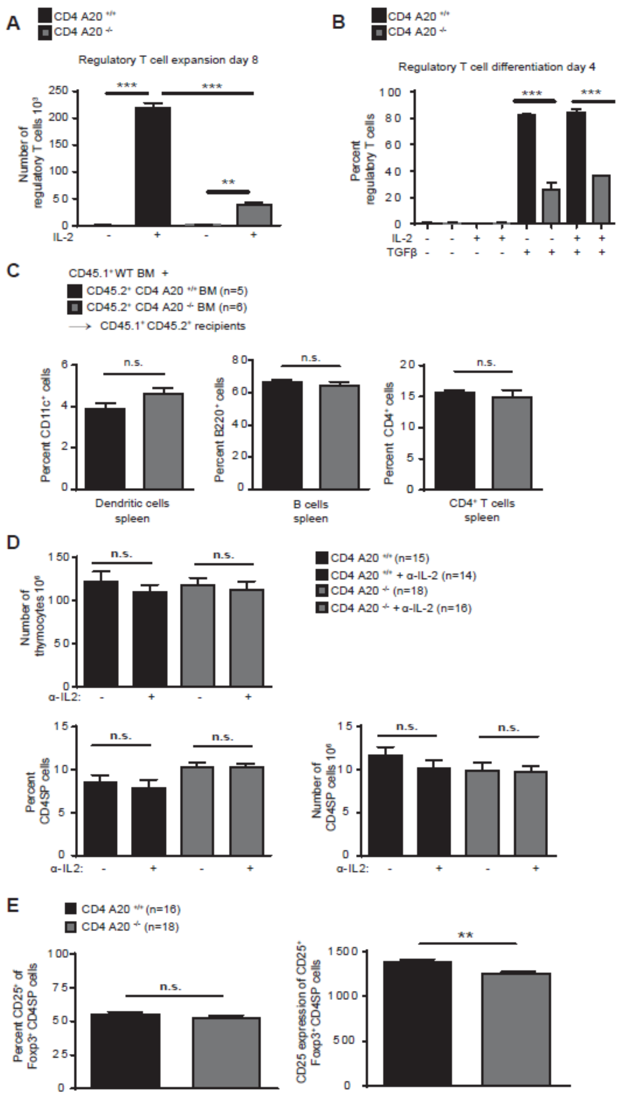

Supplementary Figure 2

Supplementary Figure 2: A20-deficient T_{reg} cells show reduced dependence on IL-2

A) 50 x 10^3 MACS-sorted $A20^{+/+}$ and $A20^{-/-}$ $CD4^+$ $CD25^+$ T_{reg} cells were cultured *in vitro* in the presence of microbeads loaded with α-CD3 and α-CD28 with or without IL-2 for 8 days and absolute numbers of live $Foxp3^+$ T_{reg} cells were determined. The experiment was performed once. **B)** 40 x 10^3 $CD4^+$ $CD62L^{high}$ $CD44^{low}$ $A20^{+/+}$ and $A20^{-/-}$ T cells were cultured *in vitro* in the presence of plate-bound α-CD3 and soluble α-CD28 +/- IL-2 and +/- TGFβ for 4 days. Conversion towards a T_{reg} cell phenotype was determined by intracellular Foxp3 staining. One representative of three independent experiments is shown. **C)** $CD45.1^+$ $CD45.2^+$ double positive C57BL/6 recipient mice received 11 Gy TBI and were transplanted with 2.5x10^6 T cell-depleted C57BL/6 WT bone marrow expressing only CD45.1. Additionally, recipient mice received either 2.5x10^6 $A20^{+/+}$ or 2.5x10^6 $A20^{-/-}$ T cell-depleted C57BL/6 bone marrow, both expressing only CD45.2. Mice were analyzed three months after transplantation by FACS. Frequencies of $CD11c^+$ dendritic cells, $B220^+$ B cells and $CD4^+$ T cells of live splenocytes are shown. The experiment was performed once. **D)** CD4 $A20^{+/+}$ or CD4 $A20^{-/-}$ mice were injected intraperitoneally with α-IL-2 neutralizing antibody and analyzed six days after first treatment. One group of each genotype was left without α-IL-2 treatment as control. Thymocytes were counted and stained with anti-CD4, anti-CD8 and live/dead reagent and population of $CD4^+$ $CD8^-$ (CD4SP) cells of all live cells was determined by flow cytometry. Upper panel: Total thymic cellularity. Lower panel: Frequency and numbers of CD4SP cells. Pooled data of three independent experiments. Animal numbers per group (n) are depicted. **E)** Thymic $CD4^+$ $CD8^-$ $Foxp3^+$ T_{reg} cells of CD4 $A20^{+/+}$ or $A20^{-/-}$ mice were analyzed for CD25 (IL-2RA) expression. Left panel: Percentages of $CD25^+$ of CD4SP $Foxp3^+$ T_{reg} cells. Right panel: Mean fluorescence intensity (MFI) of $CD25^+$ of CD4SP $Foxp3^+$ T_{reg} cells. Pooled data of three independent experiments. Animal numbers per group (n) are depicted. Experiments were analyzed using two-tailed unpaired t test. Significance was set at p values < 0.05, p < 0.01 and p < 0.001 and was then indicated with asterisks (*, ** and ***). Data are presented as mean ± S.E.M.

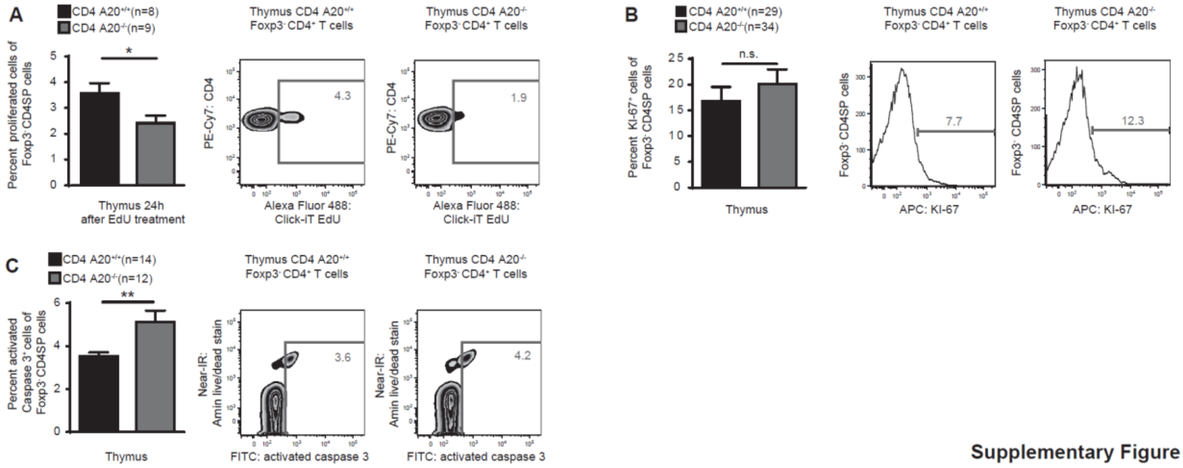

Supplementary Figure 3

Supplementary Figure 3: Thymic A20-deficient Foxp3$^-$ CD4$^+$ T cells show reduced proliferation and increased apoptosis

A) CD4 A20$^{+/+}$ and CD4 A20$^{-/-}$ mice were treated with 50 mg/kg EdU. 24h after application harvested thymocytes were stained with live/dead reagent, anti-CD4, anti-CD8, anti-Foxp3 and EdU Click-iT-® staining was performed. The population of EdU$^+$ cells of all Foxp3$^-$ CD4$^+$ CD8$^-$ (CD4SP) cells was determined by flow cytometry. Pooled data of two independent experiments are shown (left panel). Animal numbers per group (n) are depicted. Gating strategy and representative FACS plots of the experiments (right panel). **B)** Thymocytes of adult CD4 A20$^{+/+}$ and CD4 A20$^{-/-}$ mice were stained as in A and additionally stained with anti-KI-67. The population of KI-67$^+$ cells of all Foxp3$^-$ CD4SP cells was determined by flow cytometry. Pooled data of six independent experiments are shown (left panel). Animal numbers per group (n) are depicted. Gating strategy and representative histograms of the experiments (right panel). **C**) Thymocytes of adult CD4 A20$^{+/+}$ and CD4 A20$^{-/-}$ mice were incubated with an active caspase 3 staining reagent for 45min and stained as in A. The population of activated caspase 3$^+$ cells of all Foxp3$^-$ CD4SP cells was determined by flow cytometry. Pooled data of three independent experiments are shown (left panel). Animal numbers per group (n) are depicted. Gating strategy and representative FACS plots of the experiments (right panel). Experiments were analyzed using two-tailed unpaired t test. Significance was set at p values < 0.05, $p < 0.01$ and $p < 0.001$ and was then indicated with asterisks (*, ** and ***). Data are presented as mean ± S.E.M.

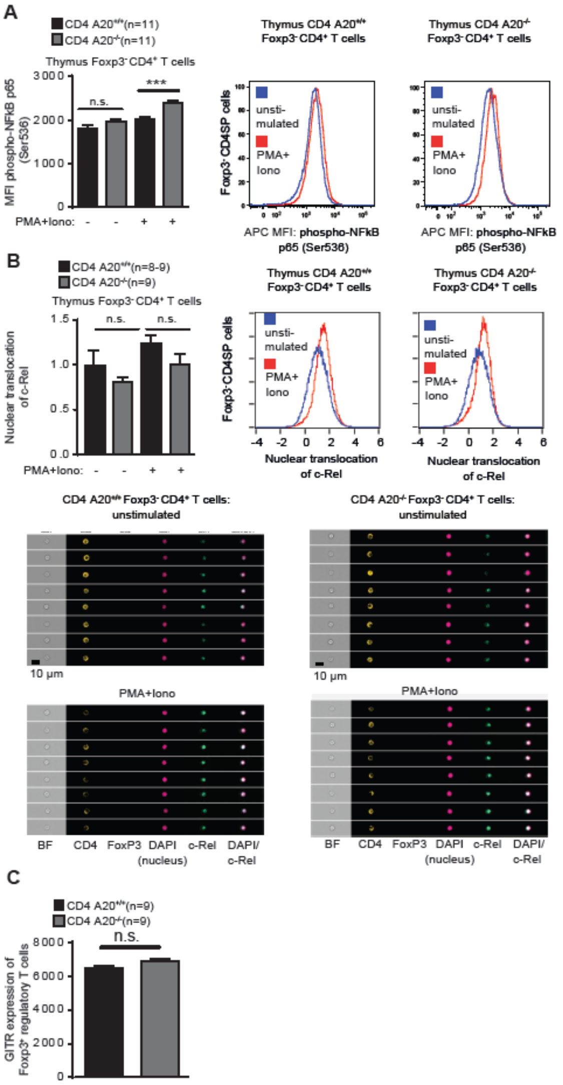

Supplementary Figure 4

Supplementary Figure 4: Thymic A20-deficient Foxp3$^-$ CD4$^+$ T cells show increased RelA activation and reduced nuclear translocation of c-Rel

A) Thymocytes of adult CD4 A20$^{+/+}$ and CD4 A20$^{-/-}$ mice were left unstimulated or stimulated with Phorbol 12-myristate 13-acetate (PMA) and Ionomycin (Iono) for 30 minutes and stained with live/dead reagent, anti-CD4, anti-CD8, anti-Foxp3 and anti-phospho-NFκB p65. The population of CD4$^+$ CD8$^-$ (CD4SP) Foxp3$^-$ cells was determined by flow cytometry and median fluorescence intensity (MFI) of phospho-NFκB p65 was calculated. Pooled data of two independent experiments are shown. Animal numbers per group (n) are depicted (left panel). Representative histograms showing the fluorescence intensity of phospho-NFκB p65 of CD4SP Foxp3$^-$ cells (right panel). **B)** CD8$^+$ cell MACS-depleted thymocytes of CD4 A20$^{+/+}$ and CD4 A20$^{-/-}$ mice were left unstimulated or stimulated with PMA and Iono for 30 minutes, stained with anti-CD4, anti-CD8, anti-Foxp3, anti-c-Rel and DAPI and acquired on an imaging flow cytometer. Nuclear translocation of CD4SP Foxp3$^-$ T_{reg} cells was quantified based on the similarity score of c-Rel and nuclear image intensities. Pooled data of two independent experiments are shown. Animal numbers per group (n) are depicted (left panel). Representative histograms show the nuclear translocation score of unstimulated and stimulated CD4SP Foxp3$^-$ T_{reg} cell populations (right panel). Exemplary images are representative for the mean nuclear translocation score of indicated populations (lower panel). **C)** Thymocytes of adult CD4 A20$^{+/+}$ and CD4 A20$^{-/-}$ mice were stained with anti-CD4, anti-CD8, anti-CD25, anti-Foxp3, anti-GITR and live/dead reagent. Mean GITR expression of Foxp3$^+$ CD4$^+$ CD8$^-$ live cells was determined by flow cytometry. Pooled data of 2 independent experiments are shown. Animal numbers per group (n) are depicted. Experiments were analyzed using two-tailed unpaired t test. Significance was set at p values < 0.05, $p < 0.01$ and $p < 0.001$ and was then indicated with asterisks (*, ** and ***). Data are presented as mean ± S.E.M.
